# The Physique of Boys

**Published:** 1909-03

**Authors:** Bertram M. H. Rogers

**Affiliations:** Physician to the Royal Hospital for Sick Children and Women; Physician to the Handel Cossham Memorial Hospital; Medical Officer to the Clifton Wood Industrial School


### THE PHYSIQUE OF BOYS.

BY

BERTRAM M. H. ROGERS, M.D., B.Ch., B.A. Oxon.,

*Physician to the Royal Hospital for Sick Children and Women;*
*Physician to the Handel Cossham Memorial Hospital;*
*Medical Officer to the Clifton Wood Industrial School.*

THE full title of this paper should be, " A comparative study of the heights, weights, and chest measurements of boys of 13, 14 and 15 years of age in Industrial and Public Schools."

For some years quarterly weighings and measurements have been made of all the boys under my care at the Clifton Wood Industrial School. This was done for three objects: (1) to have an accurate record, (2) to find if the dieting was sufficient, and (3) to show whether any boy was, unobserved, developing any tubercular disease.

As the members of this Society probably know very little about what are known as the Home Office Schools, I will, with your permission, give a few facts about these excellent institutions. The common idea is that they are supported by charity. This is not so. A few donations are made, but the bulk of the money for education, feeding and house expenses comes from two sources, the rates and the Government. I need not go into the details of this, but I may say that the schools are under a dual control, inspectors being sent by the Home Office, while a member of the Committee is specially told off to watch the boys of any particular town that sends boys to the school. In our school the majority of boys come from London, Derby and Bristol, not 5 per cent. coming from other places.

The boys are drawn from the lowest class of our civilisation; but we contend that if you catch them early, we can, like Dr. Johnson's Scotchman, do something with them. I was only the other day looking through the entry book and noting the parentage. I should think, though I cannot speak from figures, that a large percentage are the sons of prostitutes or dissolute mothers. In many cases it is recorded that the father was a drunkard, sometimes both parents. In fact, we must admit that the material we have to work on is the worst that can be obtained.

The boys themselves come to us with a bad record. They have committed some petty theft, picked a pocket, have been incorrigible truants, or possibly simply because they are living in a house where vice and drunkenness are rampant. Brought up under such conditions, it is not surprising that, mentally and physically, they are below the standard required to make good citizens. Fortunately we can remedy one of their failures. We can, and do, turn them out well educated for their station in life, and obtain for them places where they can, and do, become useful members of our country ; but we cannot, as I shall show, bring them up to the standard of those boys who have been born in a more well-to-do position.

The quarterly weighings are begun directly the boy enters the school, and the book is open for my inspection at any time. A look through has often proved of great use to me. A boy is noticed not to be gaining weight in proper proportion, or is even losing. I have him sent for. If no cause can be found, I order special diet or medicine, or suggest a month or two in the cooking department, and I have often been gratified with the result. In one case I had a boy weighed weekly for over a year. He had been under me for tubercular peritonitis in the Children's Hospital, and the weekly report was of the greatest value. He is still in the school, and doing well.

I mentioned that one of the reasons for recording weights, &c., was to see if the dieting was sufficient. I would ask you to remember that with these boys a scale of dieting has to be made, and a boy of $x$ years is supposed to eat $y$ amount of food. The rule is not absolute, but it works well. Feeding a large number can be done extremely cheap; but it will surprise you to hear that our boys are well fed on under 5d. a day, four meals being given, with meat once a day except one day of the week. The boys have to do all the manual work of the building—the washing, cleaning, and making beds—besides drill, gymnasium, carpentering, and making all their own clothes. Besides this, they play football or cricket twice a week, learn to swim, and have to walk to the Downs for their games. You will therefore see that a great deal is taken out of them. The question was were we putting enough in, and from our physique record we came to the conclusion that we were not. So we gave another meal—a glass of milk and some biscuits—with very satisfactory results. It meant an outlay of £20 or more a year, for which we received nothing from the authorities.

To come to my tables. I have always said that statistics, especially in medical matters, were futile things ; so to show my inconsistency, I am going to give you some.

These averages were worked out on a fairly large number of boys; and for the sake of comparison I have put down the standard published by Roberts, and compared the figures with those obtained from Marlborough College, the only Public School where accurate measurements have been systematically made.

The first point that must strike us in looking at these tables is that there is no recognised standard, or perhaps that the standards are inconsistent. Compare, for instance, Roberts's with that of the Anthropometric. For a boy of 13 the former gives the height as 55 inches, the latter as 57, while neither the two Industrial Schools nor Marlborough College come up to the latter, the Industrial Schools being 2 inches below. Then in weights Roberts, at the same age, gives 78 lb., the Anthropometric 82. The Industrial Schools are much below, while Marlborough College is a long way above. Even in Boulton's table, in which ages are not given, a boy of 55 inches should weigh only 77 lb., a pound less than Roberts makes out.

I need hardly comment on these tables, as a glance through them will explain the discrepancies at once. But we see the enormous advantage the boys of the better-to-do classes have. This has been explained by the better food. The food a Public School boy takes depends on two factors—the diet supplied by his house-master, and the pocket-money supplied by the parent. One point I may call attention to. The Industrial School boy starts 2 inches below the average (Roberts's) at 13, and at 15 is 3 or 2½ below, while his weight increases in greater proportion, showing that in spite of all we can do he not only does not pick up the lost ground in his height, he even loses.

In the second table I have analysed the schools for different towns. Here it is flattering to our city to observe that the Bristol boys have the best physique; but on the other hand, I am told by the masters that the London boys are more intelligent and quicker in their work.

My London figures for the Clifton School and Nautical School correspond very closely. Those for Sheffield were taken from an article in *The Practitioner*, and were made from deficient children. The author of this article says, half in apology for the high figures, that it does not follow that a mentally deficient child is physically stunted. The table shows that they are below the average of London, Bristol or Derby.

The following tables give the height, weight, and chest measurements at the ages of 13, 14, and 15. In the first the different schools are compared with the standard authorities, and in the second the comparisons are with the boys coming from various cities.

TABLE I.

| | Age 13. | | | Age 14. | | | Age 15. | | |
|---|---|---|---|---|---|---|---|---|---|
| | H. | W. | CH. | H. | W. | CH. | H. | W. | CH. |
| Roberts .. .. .. | 55 | 78 | 25 | 58 | 84 | 26 | 60.5 | 94 | 27 |
| Anthropomet. Stand. | 57 | 82 | — | 59 | 91 | — | 61 | 102 | — |
| Boulton (no age) .. | 55 | 77 | — | 59 | 91 | — | 60 | 90 | — |
| Clifton Wood .. | 53 | 71.5 | 26.7 | 54.9 | 83.5 | 27 | 57.5 | 83.9 | 28.6 |
| Nautical School .. | 53 | 71.9 | 25.8 | 55 | 81.5 | 27.2 | 57 | 98.2 | 28.9 |
| Marlboro' College .. | 56 | 95 | 31.1 | 61 | 105 | 32.2 | 63 | 120 | 33.4 |

TABLE II.

| | Age 13. | | | Age 14. | | | Age 15. | | |
|---|---|---|---|---|---|---|---|---|---|
| | H. | W. | CH. | H. | W. | CH. | H. | W. | CH. |
| London .. .. .. .. | 51.7 | 67 | 26.1 | 53.4 | 76.4 | 27 | 55.5 | 83 | 28 |
| Derby .. .. .. .. | 53 | 71.5 | 26.6 | 55 | 89.9 | 27.4 | 57.2 | 92 | 28.8 |
| Bristol .. .. .. .. | 54.5 | 75 | 27.5 | 56.3 | 84.3 | 28.4 | 60 | 90.6 | 28.8 |
| Sheffield .. .. | 51 | 60 | — | 55 | 76 | — | 57.5 | 77 | — |
| London, N.S. .. | 52.6 | 71 | 25.8 | 55 | 77.8 | 27.3 | 54 | 83.5 | 28.5 |

